# Supplementary material for: Hot spot prediction in protein-protein interactions by an ensemble system
Source: BMC Syst Biol. 2018 Dec 31;12(Suppl 9):132. doi: 10.1186/s12918-018-0665-8 (PMC6311905; doi:10.1186/s12918-018-0665-8)
Supplement: Supplementary file 2 — All relevant MATLAB code. A simple Matlab implement of our predictor. (ZIP 19,742 kb) [file 12918_2018_665_MOESM2_ESM.zip › Additional file 2/Read ASA/ASA website/dbMPIKT/1K8RA.html]

 NetSurfP - Protein Surface Accessibility and Secondary Structure Predictions

  

|  |  |  |
| --- | --- | --- |
|  |  | NetSurfP - Protein Surface Accessibility and Secondary Structure Predictions  Technical University of Denmark |

  


---

```
# For publication of results, please cite:
# A generic method for assignment of reliability scores applied to solvent accessibility predictions.
# Bent Petersen, Thomas Nordahl Petersen, Pernille Andersen, Morten Nielsen and Claus Lundegaard
# BMC Structural Biology 2009, 9:51 doi:10.1186/1472-6807-9-51
#
# Column 1: Class assignment - B for buried or E for Exposed - Threshold: 25% exposure, but not based on RSA
# Column 2: Amino acid
# Column 3: Sequence name
# Column 4: Amino acid number
# Column 5: Relative Surface Accessibility - RSA
# Column 6: Absolute Surface Accessibility
# Column 7: Z-fit score for RSA prediction
# Column 8: Probability for Alpha-Helix
# Column 9: Probability for Beta-strand
# Column 10: Probability for Coil
E M  1K8RA                  1    0.601 120.240  -0.853   0.003   0.003   0.994
E T  1K8RA                  2    0.458  63.525   1.259   0.004   0.085   0.910
E E  1K8RA                  3    0.440  76.885   1.842   0.004   0.514   0.481
B Y  1K8RA                  4    0.078  16.669   1.004   0.002   0.816   0.182
B K  1K8RA                  5    0.174  35.854   1.519   0.001   0.900   0.099
B L  1K8RA                  6    0.031   5.639   1.098   0.000   0.983   0.017
B V  1K8RA                  7    0.023   3.597   1.392   0.000   0.983   0.017
B V  1K8RA                  8    0.019   2.936   1.405   0.001   0.959   0.040
B V  1K8RA                  9    0.026   3.996   0.912   0.001   0.900   0.099
B G  1K8RA                 10    0.040   3.156  -0.573   0.004   0.420   0.576
B A  1K8RA                 11    0.197  21.731  -0.571   0.005   0.045   0.951
B G  1K8RA                 12    0.260  20.486  -1.382   0.018   0.019   0.964
B G  1K8RA                 13    0.213  16.755  -1.337   0.115   0.016   0.868
B V  1K8RA                 14    0.052   8.023   0.005   0.339   0.016   0.645
B G  1K8RA                 15    0.042   3.305  -1.320   0.430   0.016   0.555
B K  1K8RA                 16    0.092  18.822  -0.055   0.802   0.014   0.185
B S  1K8RA                 17    0.052   6.118  -0.083   0.879   0.010   0.111
B A  1K8RA                 18    0.033   3.681  -0.259   0.938   0.007   0.055
B L  1K8RA                 19    0.031   5.639   0.355   0.975   0.003   0.022
B T  1K8RA                 20    0.022   3.079   0.604   0.975   0.003   0.022
B I  1K8RA                 21    0.112  20.683   0.585   0.938   0.007   0.055
B Q  1K8RA                 22    0.104  18.574   1.146   0.938   0.007   0.055
B L  1K8RA                 23    0.070  12.835   0.198   0.938   0.007   0.055
B I  1K8RA                 24    0.062  11.562   0.382   0.802   0.014   0.185
E Q  1K8RA                 25    0.507  90.550   0.912   0.600   0.003   0.397
E N  1K8RA                 26    0.592  86.742  -0.131   0.176   0.004   0.820
E H  1K8RA                 27    0.457  83.074   1.355   0.113   0.043   0.844
B F  1K8RA                 28    0.061  12.303   0.066   0.053   0.043   0.903
E V  1K8RA                 29    0.388  59.605   1.631   0.053   0.043   0.903
E D  1K8RA                 30    0.707 101.951   0.101   0.053   0.043   0.903
E E  1K8RA                 31    0.645 112.612  -0.049   0.053   0.043   0.903
B Y  1K8RA                 32    0.225  48.018   0.595   0.052   0.084   0.864
E D  1K8RA                 33    0.528  76.085   0.802   0.052   0.084   0.864
B P  1K8RA                 34    0.166  23.570   0.007   0.052   0.084   0.864
B T  1K8RA                 35    0.144  19.903   0.069   0.148   0.418   0.435
B I  1K8RA                 36    0.122  22.644   0.671   0.079   0.592   0.329
B E  1K8RA                 37    0.173  30.258  -0.663   0.074   0.484   0.442
B D  1K8RA                 38    0.143  20.621  -0.071   0.087   0.683   0.230
B S  1K8RA                 39    0.264  30.953  -0.583   0.021   0.756   0.223
B Y  1K8RA                 40    0.095  20.259   0.430   0.001   0.900   0.099
E R  1K8RA                 41    0.359  82.188   1.358   0.001   0.959   0.040
B K  1K8RA                 42    0.237  48.854   1.610   0.001   0.959   0.040
E Q  1K8RA                 43    0.398  71.047   1.622   0.000   0.983   0.017
B V  1K8RA                 44    0.064   9.898   1.310   0.000   0.983   0.017
E V  1K8RA                 45    0.495  76.035   0.702   0.000   0.983   0.017
B I  1K8RA                 46    0.110  20.276   0.728   0.001   0.959   0.040
E D  1K8RA                 47    0.689  99.256   0.797   0.004   0.138   0.858
E G  1K8RA                 48    0.795  62.551   0.171   0.003   0.003   0.994
E E  1K8RA                 49    0.540  94.338   1.366   0.003   0.718   0.279
E T  1K8RA                 50    0.451  62.609   2.021   0.001   0.959   0.040
B C  1K8RA                 51    0.045   6.360   1.284   0.006   0.962   0.032
E L  1K8RA                 52    0.296  54.161   2.455   0.006   0.962   0.032
B L  1K8RA                 53    0.025   4.578   1.044   0.001   0.959   0.040
B D  1K8RA                 54    0.116  16.716   1.119   0.011   0.918   0.071
B I  1K8RA                 55    0.026   4.791   0.908   0.011   0.918   0.071
B L  1K8RA                 56    0.088  16.131   0.954   0.018   0.846   0.136
B D  1K8RA                 57    0.086  12.465   0.281   0.022   0.552   0.426
B T  1K8RA                 58    0.123  17.032   0.266   0.056   0.142   0.802
B A  1K8RA                 59    0.151  16.585  -0.411   0.115   0.016   0.868
E G  1K8RA                 60    0.279  21.973  -0.841   0.257   0.016   0.727
B Q  1K8RA                 61    0.247  44.078   0.298   0.522   0.016   0.462
E E  1K8RA                 62    0.373  65.198  -0.289   0.622   0.015   0.363
E E  1K8RA                 63    0.371  64.779   0.621   0.660   0.049   0.291
B Y  1K8RA                 64    0.091  19.404   0.177   0.660   0.049   0.291
E S  1K8RA                 65    0.398  46.646   1.096   0.660   0.049   0.291
B A  1K8RA                 66    0.207  22.866  -1.062   0.522   0.016   0.462
B M  1K8RA                 67    0.106  21.171   0.329   0.522   0.016   0.462
B R  1K8RA                 68    0.181  41.449   0.510   0.522   0.016   0.462
E D  1K8RA                 69    0.480  69.197  -0.278   0.522   0.016   0.462
B Q  1K8RA                 70    0.230  41.114  -0.267   0.561   0.047   0.393
B Y  1K8RA                 71    0.161  34.470  -0.554   0.502   0.102   0.396
B M  1K8RA                 72    0.100  19.990   0.258   0.428   0.171   0.402
B R  1K8RA                 73    0.284  64.944   0.241   0.191   0.086   0.723
E T  1K8RA                 74    0.370  51.361  -0.592   0.053   0.043   0.903
B G  1K8RA                 75    0.037   2.880  -0.656   0.019   0.141   0.840
B E  1K8RA                 76    0.176  30.730   0.140   0.005   0.336   0.660
B G  1K8RA                 77    0.023   1.786   0.636   0.002   0.816   0.182
B F  1K8RA                 78    0.019   3.813   1.298   0.001   0.959   0.040
B L  1K8RA                 79    0.017   3.168   1.645   0.001   0.959   0.040
B C  1K8RA                 80    0.018   2.513   1.745   0.000   0.983   0.017
B V  1K8RA                 81    0.023   3.458   1.178   0.001   0.959   0.040
B F  1K8RA                 82    0.059  11.761   1.077   0.001   0.959   0.040
B A  1K8RA                 83    0.068   7.527  -0.514   0.002   0.816   0.182
B I  1K8RA                 84    0.059  10.841   0.687   0.005   0.336   0.660
E N  1K8RA                 85    0.282  41.358   0.247   0.005   0.045   0.951
E N  1K8RA                 86    0.352  51.474   0.935   0.018   0.019   0.964
E T  1K8RA                 87    0.443  61.500   1.524   0.694   0.003   0.303
E K  1K8RA                 88    0.589 121.198   1.370   0.600   0.003   0.397
B S  1K8RA                 89    0.059   6.880   0.216   0.694   0.003   0.303
B F  1K8RA                 90    0.064  12.845  -0.139   0.923   0.002   0.076
E E  1K8RA                 91    0.584 102.077   1.577   0.970   0.001   0.030
E D  1K8RA                 92    0.371  53.519   1.524   0.970   0.001   0.030
B I  1K8RA                 93    0.017   3.127   1.340   0.970   0.001   0.030
E H  1K8RA                 94    0.461  83.820   1.658   0.988   0.000   0.012
E Q  1K8RA                 95    0.483  86.210   2.276   0.970   0.001   0.030
B Y  1K8RA                 96    0.039   8.377   0.655   0.988   0.000   0.012
B R  1K8RA                 97    0.113  25.785   1.416   0.988   0.000   0.012
E E  1K8RA                 98    0.590 102.986   2.084   0.988   0.000   0.012
B Q  1K8RA                 99    0.213  38.042   1.544   0.988   0.000   0.012
B I  1K8RA                100    0.013   2.442   1.592   0.970   0.001   0.030
E K  1K8RA                101    0.374  76.891   1.811   0.975   0.003   0.022
E R  1K8RA                102    0.682 156.270   1.553   0.938   0.007   0.055
E V  1K8RA                103    0.229  35.228   0.650   0.782   0.003   0.216
B K  1K8RA                104    0.244  50.088  -0.040   0.321   0.003   0.675
E D  1K8RA                105    0.713 102.801  -0.393   0.053   0.005   0.942
E S  1K8RA                106    0.400  46.892  -0.412   0.016   0.005   0.979
E D  1K8RA                107    0.741 106.778  -0.719   0.018   0.019   0.964
E D  1K8RA                108    0.486  70.004   0.996   0.005   0.015   0.979
B V  1K8RA                109    0.030   4.580   0.980   0.004   0.197   0.799
B P  1K8RA                110    0.093  13.154   0.885   0.002   0.816   0.182
B M  1K8RA                111    0.034   6.823   0.864   0.001   0.959   0.040
B V  1K8RA                112    0.015   2.305   1.750   0.000   0.983   0.017
B L  1K8RA                113    0.014   2.600   1.736   0.001   0.959   0.040
B V  1K8RA                114    0.015   2.290   1.442   0.001   0.900   0.099
B G  1K8RA                115    0.023   1.786   0.065   0.004   0.616   0.381
B N  1K8RA                116    0.077  11.331  -0.480   0.022   0.359   0.619
B K  1K8RA                117    0.177  36.429   0.201   0.019   0.141   0.840
B C  1K8RA                118    0.163  22.857  -0.088   0.018   0.047   0.935
B D  1K8RA                119    0.223  32.120   0.025   0.018   0.047   0.935
B L  1K8RA                120    0.221  40.520   0.223   0.058   0.017   0.925
E A  1K8RA                121    0.671  73.999   0.693   0.058   0.017   0.925
E A  1K8RA                122    0.688  75.829   0.651   0.058   0.017   0.925
E R  1K8RA                123    0.270  61.830   0.998   0.053   0.043   0.903
E T  1K8RA                124    0.564  78.255   1.047   0.056   0.142   0.802
B V  1K8RA                125    0.060   9.283   0.692   0.018   0.088   0.893
E E  1K8RA                126    0.414  72.256   1.538   0.018   0.047   0.935
E S  1K8RA                127    0.484  56.748   1.222   0.923   0.002   0.076
E R  1K8RA                128    0.688 157.438   1.243   0.970   0.001   0.030
E Q  1K8RA                129    0.345  61.635   1.687   0.970   0.001   0.030
B A  1K8RA                130    0.027   2.997  -0.064   0.970   0.001   0.030
E Q  1K8RA                131    0.370  66.118   2.156   0.970   0.001   0.030
E D  1K8RA                132    0.567  81.734   1.957   0.970   0.001   0.030
B L  1K8RA                133    0.099  18.200   1.275   0.970   0.001   0.030
B A  1K8RA                134    0.018   2.028   0.098   0.970   0.001   0.030
E R  1K8RA                135    0.717 164.101   1.545   0.970   0.001   0.030
E S  1K8RA                136    0.641  75.149   1.351   0.858   0.002   0.139
E Y  1K8RA                137    0.265  56.652   1.463   0.406   0.004   0.590
E G  1K8RA                138    0.777  61.181   0.721   0.003   0.003   0.994
B I  1K8RA                139    0.047   8.677   0.552   0.005   0.015   0.979
E P  1K8RA                140    0.312  44.315   1.742   0.005   0.336   0.660
B Y  1K8RA                141    0.078  16.690   0.536   0.001   0.900   0.099
B I  1K8RA                142    0.081  14.948   0.398   0.011   0.918   0.071
B E  1K8RA                143    0.066  11.478   0.124   0.011   0.918   0.071
B T  1K8RA                144    0.031   4.272   0.197   0.021   0.756   0.223
B S  1K8RA                145    0.067   7.806  -0.022   0.021   0.451   0.528
B A  1K8RA                146    0.044   4.827  -1.321   0.125   0.227   0.648
B K  1K8RA                147    0.245  50.314   0.387   0.113   0.043   0.844
E T  1K8RA                148    0.476  65.980   0.331   0.058   0.017   0.925
E R  1K8RA                149    0.525 120.156  -0.622   0.109   0.005   0.886
E Q  1K8RA                150    0.447  79.763   1.246   0.321   0.003   0.675
B G  1K8RA                151    0.243  19.116   0.989   0.181   0.016   0.803
B V  1K8RA                152    0.019   2.997   1.511   0.923   0.002   0.076
E E  1K8RA                153    0.391  68.308   2.212   0.970   0.001   0.030
E D  1K8RA                154    0.439  63.289   2.066   0.970   0.001   0.030
B A  1K8RA                155    0.030   3.350   1.187   0.988   0.000   0.012
B F  1K8RA                156    0.022   4.395   1.300   0.988   0.000   0.012
E Y  1K8RA                157    0.324  69.282   2.269   0.970   0.001   0.030
E T  1K8RA                158    0.338  46.867   2.257   0.970   0.001   0.030
B L  1K8RA                159    0.030   5.511   1.043   0.988   0.000   0.012
B V  1K8RA                160    0.031   4.811   0.235   0.988   0.000   0.012
E R  1K8RA                161    0.435  99.523   2.108   0.988   0.000   0.012
E E  1K8RA                162    0.271  47.431   1.863   0.970   0.001   0.030
B I  1K8RA                163    0.029   5.384   0.132   0.923   0.002   0.076
E R  1K8RA                164    0.356  81.570   1.578   0.858   0.002   0.139
E Q  1K8RA                165    0.753 134.414   1.648   0.694   0.003   0.303
E H  1K8RA                166    0.706 128.385   0.478   0.016   0.005   0.979

Explain the output.  Go back.
```

---
